# Engaging Mind, Body, and Spirit: Less Isolation Through More Stimulation With YUMMY Time

**DOI:** 10.1093/geroni/igaa057.2310

**Published:** 2020-12-16

**Authors:** Connie Corley, Spring Groove

**Affiliations:** 1 Fielding Graduate University, San Gabriel, California, United States; 2 Y.U.M.M.Y. Time, Los Angeles, California, United States

## Abstract

Loneliness and social isolation in community-residing older adults is drawing increasing attention. Yet the needs of nursing home/assisted living residents is critical as well. Engaging professionally-trained musicians who are certified yoga instructors in providing a structured, yet flexible, program offered regularly in such settings is an innovation meriting examining what is observed as a profound experience for both older adults and care providers. Y.U.M.M.Y. Time (through music, meditation and yoga) is a paradigm created by Spring Groove (Gross) offering a unique intersection between the performance arts and the contemplative arts. We are studying the benefits of this approach combining sensory stimulation with inner engagement, with the goal to expand into community settings. As staff members note: “Although the ingredients are simple, the results are profound.” “The program is very enriching, full of great music moments, yoga and meditation to relax our residents. It is very much enjoyed by everyone!”

